# Design of a High *Q*-Factor Label-Free Optical Biosensor Based on a Photonic Crystal Coupled Cavity Waveguide

**DOI:** 10.3390/s24010193

**Published:** 2023-12-28

**Authors:** Reyhaneh Jannesari, Gerald Pühringer, Gerald Stocker, Thomas Grille, Bernhard Jakoby

**Affiliations:** 1 Institute for Microelectronics and Microsensors, Johannes Kepler University, 4040 Linz, Austria; gerald.puehringer@jku.at (G.P.); bernhard.jakoby@jku.at (B.J.); 2 Infineon Technologies Austria AG, 9520 Villach, Austriathomas.grille@infineon.com (T.G.)

**Keywords:** photonic crystals, label-free biosensor, coupled cavity waveguide, photonic band gap, refractive index sensor

## Abstract

In recent years, there has been a significant increase in research into silicon-based on-chip sensing. In this paper, a coupled cavity waveguide (CCW) based on a slab photonic crystal structure was designed for use as a label-free biosensor. The photonic crystal consisted of holes arranged in a triangular lattice. The incorporation of defects can be used to design sensor devices, which are highly sensitive to even slight alterations in the refractive index with a small quantity of analyte. The plane wave expansion method (PWE) was used to study the dispersion and profile of the CCW modes, and the finite difference time domain (FDTD) technique was used to study the transmission spectrum, quality factor, and sensitivity. We present an analysis of adiabatically coupling light into a coupled cavity waveguide. The results of the simulation indicated that a sensitivity of 203 nm/RIU and a quality factor of 13,360 could be achieved when the refractive indices were in the range of 1.33 to 1.55.

## 1. Introduction

Biosensing is a crucial technology in many fields of application, including environment protection, food and water monitoring, clinical diagnostics, and healthcare [1,2,3]. A key issue in this area is to find ways to enhance the sensitivity and accuracy of smaller devices [4,5]. Among the different fields of biosensing, optical sensors are powerful candidates to tackle most of the mentioned challenges. Optical sensors convert light into an electrical signal for a real-time analysis of a (preferably) small volume of analyte [6,7,8]. There is a wide variety of different optical sensor technologies, such as waveguiding devices, surface functionalization, and optofluidic integration. This paper investigates the concept and design of a label-free optical sensor that is based on a photonic crystal waveguide. Label-free interaction analysis techniques use optics-based biosensors to convert biological binding responses into signals without using artificial probes or labels such as fluorescent markers [9]. Therefore, this technique provides data in real time that is closer to the natural behavior of detected analytes or molecules. Photonic crystal-based biosensors have the ability to offer rapid, label-free detection of multiple substances, making diagnosis simpler, cheaper, and more efficient. In this work, the considered sensing mechanism was based on refractive index sensing, in which a device with a resonant peak is designed to convert a small change in the refractive index into an associated frequency shift of the resonant peak [6,10]. The refractive indices of various common and useful analytes in different concentrations, which are used as references for the simulations in this work, are provided in [4].

Many available biosensors currently leverage the surface plasmon resonance (SPR) effect, essentially detecting changes in the refractive index (RI) near a metal surface [11,12]. However, due to the limited penetration depth of surface plasmon (SP) modes, the probing capability of SPR sensors is confined to a few tens of nanometers above the metal surface. These sensors encounter significant challenges, including intrinsic losses and complexities in fabrication and integration with other optical or electrical components. Biosensors based on photonic crystals (PhCs) offer solutions to these issues. The interaction of a photonic crystal mode and surface plasmon modes can result in interesting optical phenomena and has often been explored for various applications in photonics [13,14,15,16]. Photonic crystals feature exceptionally narrow resonant characteristics, which can be finely tuned by the adsorption of biochemical materials. Utilizing the distinctive properties of PhCs holds the potential for more efficient and accurate biological sensing [6]. Various photonic crystal (PC) structures have been investigated and fabricated for biosensing purposes. One highly sensitive sensor was constructed from a slot PC waveguide and demonstrated a sensitivity exceeding 1150 (nm/RIU) with a measured refractive index (RI) in the range of 1 to 1.50 [17]. This sensor operates at a resonant wavelength within the mid-infrared spectrum. Painam et al. [18] introduced another PC waveguide composed of air holes in silicon with an operating wavelength of 1550 nm and a sensitivity measured at 35 nm/RIU within the refractive index range of 1.33–1.4. An alternative strategy in biosensing involves the utilization of Lx resonators with very high *Q*-factors, where ‘X’ denotes the count of removed air holes in a hexagonal network. For instance, when X = 4, results yielded a *Q*-factor of more than 8600 and a sensitivity of approximately 147 nm/RIU [19].

The abilities of photonic crystals (PhCs), such as the confinement of light and thus electromagnetic field energy in small spaces, their compatibility with waveguides, and resonators with high quality factors have sparked a great deal of interest in the development of microscale photonic integrated circuits [20]. Photonic crystals represent a periodic modification of refractive indices featuring a period in the order of the wavelength with the ability to manipulate the propagation of light. The periodic refractive index of photonic crystals results in certain frequencies of photons being able to propagate through the crystal and other frequencies being blocked, creating gaps known as band gaps [21]. Thus, no light with a frequency in the band gap can propagate within the photonic crystal. A complete band gap forbids light propagation in all directions. With the introduction of a line or point defect into the photonic crystal structure, a defect state can be created within the band gap, and this defect can be used to create a waveguide or cavity. Field profiles of associated so-called defect modes are highly confined in the waveguide or cavity area. A photonic crystal can be arranged in a periodic one-dimensional (1D), two-dimensional (2D), or three-dimensional (3D) structure. Two-dimensional (or quasi-2D) photonic crystals have become increasingly popular due to their compatibility with advanced Si-based fabrication technologies, such as the complementary metal-oxide semiconductor (CMOS) and micro-electro-mechanical systems (MEMS), which makes their fabrication process more feasible for mass production.

As discussed, a common approach to forming waveguides in PhCs is intentionally introducing “line defects” within an otherwise periodic structure. An alternate way to make a PhC waveguide is to insert a sequence of strongly confined cavities or point defects into the PhC, which is referred to as a coupled cavity waveguide (CCW). Coupled cavity waveguides are a subclass of PhC waveguides, specifically a hybridization of line and point defects [22]. The transmission of light in CCWs can be viewed as photons jumping between adjacent cavities due to the overlapping of the tightly confined modes [23]. These structures can be used to create optical delay lines, filters, routers, pulse shapers, and dispersion compensators with customized group velocity dispersion [24,25]. The uniqueness of this work lies in the innovative concept of a photonic crystal (PhC) waveguide formed by a coupled cavity waveguide (CCW). The number of coupled cavities can be adjusted to customize both the resonance modes and the waveguide’s length. Additionally, the study explores the adiabatic coupling between CCW and the photonic crystal waveguide. The design of the CCW, which achieves a high *Q*-factor, considers the avoidance of small features that may pose challenges in the fabrication process. 

## 2. Photonic Crystal and Coupled Cavity Waveguide Structure

**Figure 1:**
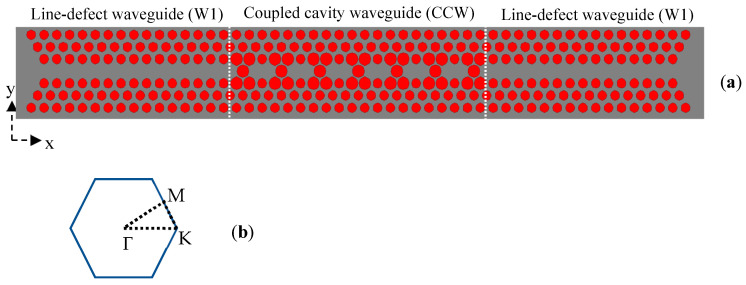
(**a**) Schematic of the structure. The grey area shows the silicon and the red circles indicate the air holes. The white dashed lines indicate the coupled cavity waveguide (CCW) area. Slab waveguides at the input and output mentioned in the text are extended from the tapered regions to the left and right, respectively. The structure has symmetrical input and output ports. (**b**) The first Brillouin zone of a triangular lattice is represented by a hexagonal shape. The vertices of this hexagon, which are referred to as high-symmetry points, have the following Cartesian coordinates: Γ(0,0), K$\left(\frac{4\pi }{3a},0\right)$, M$\left(\frac{\pi }{a},\frac{\pi }{a\sqrt{3}}\right)$.

In this work, an optical sensor based on CCWs for refractive index sensing is proposed. The adiabatic coupling technique between a PhC waveguide (W1) and a CCW in 2D PhCs is analyzed and discussed using finite difference time domain (FDTD) simulations and the plane wave expansion (PWE) method. An adiabatic transmission can take place if the operating mode of the waveguide is propagating (non-evanescent) and guided at each point of the waveguide [26,27]. This approach was employed for the effective design of a composite waveguide comprising multiple adiabatically coupled waveguide segments. Each waveguide segment efficiently guides light within the desired wavelength range. The PhC used was a triangular lattice of air holes with radii r in a silicon slab (*n* = 3.42), and the line-defect waveguide (W1) was created by removing air holes in one row in the ΓΚ direction (adopting the common notation of crystal physics, see Figure 1b). The CCW was then designed by changing the size of five air holes in the waveguide and its adjacent mirror PhC; a schematic illustration of the structure is presented in Figure 1a. The designed sensor featured symmetrical input and output ports. The fundamental mode of the silicon slab was anticipated to be coupled to the PhC waveguide mode (W1) and subsequently to the CCW mode. As depicted in Figure 1a, the sensor was designed with symmetrical structures for both input and output. However, for simplicity in understanding the concept throughout the manuscript, it is assumed that the input of the waveguide is on the left side. The tapered shape at the input (output) of the W1 waveguide was specifically designed to minimize lateral radiational loss by facilitating coupling from the fundamental slab mode to the PhC waveguide mode. A comprehensive study of this design was previously presented in [26]

**Figure 2:**
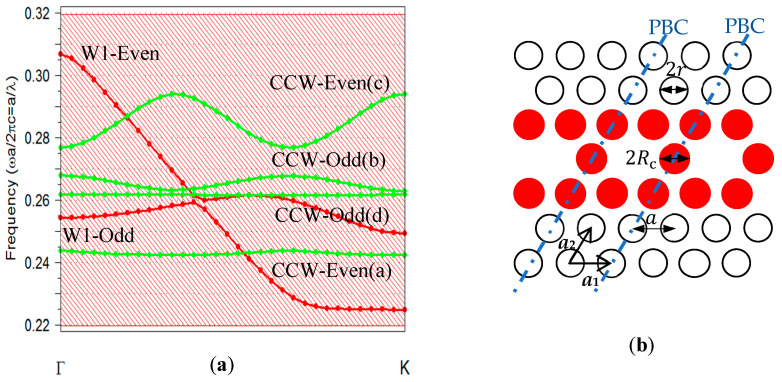
(**a**) The TE band structure of the considered CCW cavity modes with r=0.34a and $R_{c}=0.38a$ (green dotted lines) and dispersion curve of the line defect waveguide (red dotted line). The entire shadow area illustrates the photonic band gap of the unaltered crystal. (**b**) A schematic representation of the CCW; the red circles define the cavity holes with radius $R_{c}$, and the black circles define the PhC holes with radius r. Blue dotted/dashed lines define the boards of unit cells with periodic boundary conditions (PBCs).

**Figure 3:**
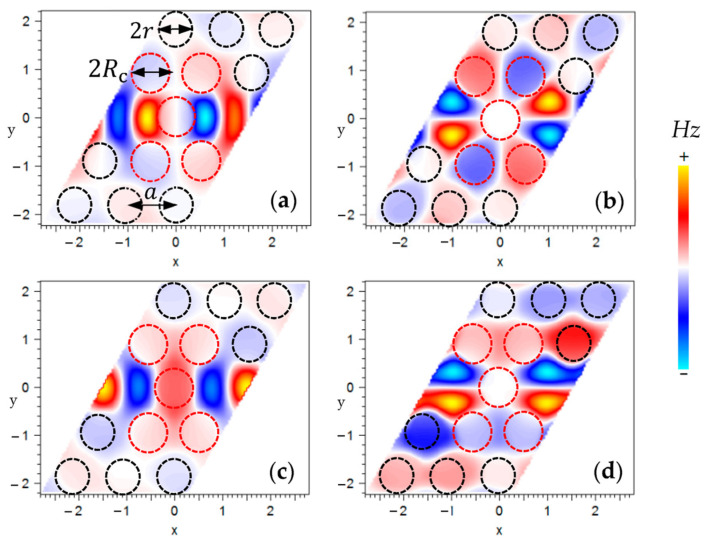
The simulated *Hz* profile in the x–y plane for a CCW with $R_{c}=0.38a$ and r=0.34a; (**a**) lower frequency even mode, (**b**) lower frequency odd mode, (**c**) higher frequency even mode, (**d**) higher frequency odd mode. Dashed circles define the positions of air holes. The modes are displayed at the Γ(0,0) in the dispersion diagram; each band is illustrated in Figure 2a.

The PWE method deals with periodic structures like photonic crystals (PhCs) because it relies on expanding periodic electromagnetic fields and permittivity functions into Fourier series. The components of these series are then analyzed using linear algebraic numerical techniques. The numerical efficiency of the PWE method is reflected in the convergence rate within the truncated basis of Fourier components. In this study, the Fourier component was limited to 64 in both the x and y directions for the 2D calculations. The structure’s geometry, the first Brillouin zone in reciprocal space, and the depiction of the periodic cell are illustrated in Figure 1a,b, and Figure 2b, respectively. Figure 2a presents the obtained dispersion diagram of the TE-coupled cavities and waveguide modes, which are characterized by electric field components that are mainly in the x–y plane. Studying the dispersion diagram shows that the coupled cavities supported four TE modes (green dotted lines), which turned out to be two even and two odd modes. On the contrary, the waveguide supported two TE modes (one even and one odd). The even resonance modes had a symmetric mode profile regarding the horizontal symmetry line of the cavity/waveguide. The odd modes had an anti-symmetric mode profile regarding the horizontal symmetry line of the cavity. The mode profiles of the CCW modes with even and odd mode symmetry for one unit cell are plotted in Figure 3a–d. The PWE method was used for this calculation [28]. The modes are displayed at the Γ(0,0) position in the first Brillouin zone.

Light was coupled into and out of the CCW using a slab waveguide followed by the W1 waveguide (Figure 1a). The waveguides were constructed in the ΓK direction of the first Brillouin zone (Figure 1b). Note that the symmetry allows, in principle, the coupling of a fundamental slab mode (i.e., the mode guided in the silicon slab without any holes), which has even symmetry to the even modes of W1 and then to the even CCW modes. Hence, antisymmetric modes do not couple to the fundamental mode of the slab waveguide, and they do not contribute to the transmittance [29,30]. Therefore, only modes with even symmetry were used for the proposed sensing application. 

## 3. Results and Discussion

**Figure 4:**
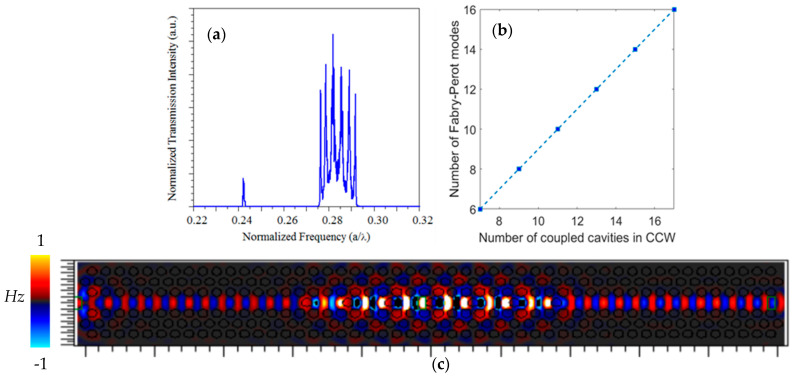
(**a**) The transmission spectra of coupling light from the slab waveguide to a W1 waveguide followed by the CCW, with $R_{c}=0.38a$ composed of seven coupled cavities, and then to the output W1 and slab waveguide. The higher frequency mode presents Fabry–Perot oscillations. (**b**) Linear relationship between the number of coupled cavities in the CCW and the number of Fabry–Perot modes. (**c**) *Hz* profile of propagating mode with normalized frequency a/λ=0.285 along the sensor. This PhC waveguide and CCW mode have even symmetry. Low lateral radiation loss in the coupling from input taper to PhC waveguide and from PhC waveguide to CCW can be seen.

A lattice constant of a=390 nm and an air hole radius r=0.34a were used as examples in this work. The CCW consisted of seven individual cavities that were horizontally coupled to create the CCW (see Figure 1a). On the basis of this structure, the normalized transmission spectrum at the output for the CCW with a cavity radius of $R_{c}=0.38a$ is shown in Figure 4a. The FDTD method was used to calculate the transmittance spectrum [28]. In this calculation, a pulse was launched into the slab waveguide, the propagating mode was coupled from the slab waveguide into the W1 waveguide followed by the CCW waveguide, then to the output W1, and finally into another slab waveguide. The resolution of all calculations was set to a rectangular grid of a/32, where a represents the lattice constant. The boundaries of the simulation area were surrounded by perfectly matched layers (PML). The transmission intensity was detected by monitoring the power intensity at the output port of the waveguide. In the normalized transmission spectrum, the resonant modes in the normalized frequency range of 0.242–0.243(a/λ) and 0.275–0.292(a/λ), corresponding to the CCW even modes in the dispersion diagram (Figure 2a), could be depicted. The comb-like shape superposed to the spectrum of the higher energy mode indicated some Fabry–Perot oscillations as a consequence of the multiple reflections from two ends of the CCW. 

To verify this concept, similar structures that had the CCW part with a greater number of coupled cavities were investigated. The longer length of the CCW resulted in Fabry–Perot oscillations with a smaller free spectral range in the same spectral region, or, in other words, a larger number of Fabry–Perot modes. Figure 4b presents the linear relation between the number of coupled cavities in the CCW and the number of Fabry–Perot modes when the size of other parts of the structure was not changed. The profile of higher frequency mode propagation at a normalized frequency, a/λ=0.285, is shown in Figure 4c. As can be seen, the propagating mode was very well localized to the waveguide area. The lateral radiational loss due to coupling light from the photonic crystal waveguide to the CCW barely can be seen. Additionally, the symmetry profile of the PhC waveguide and CCW are in good agreement with the mode profile of higher frequency even mode calculated with the PWE method (shown in Figure 3c).

**Figure 5:**
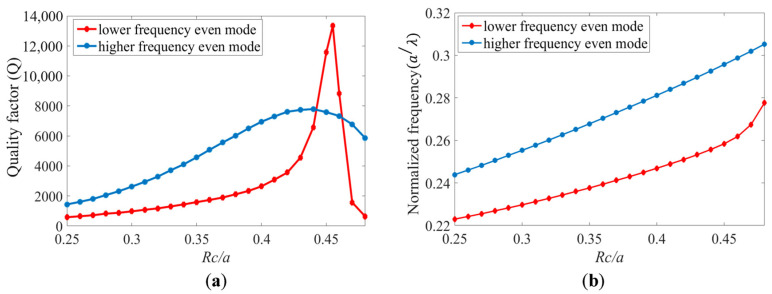
(**a**) The *Q*-factors and (**b**) resonance frequencies as a function of $R_{c}$ in the CCW structure for even modes.

Sensitivity and *Q*-factor are the most important parameters of an optical biosensor of this type. Therefore, in this work, using the FDTD simulation, we adjusted the radii of holes in the cavity, $R_{c}$ (indicated in Figure 2b), to optimize the *Q*-factor. Figure 5a presents the evaluation of the *Q*-factor of the two even cavity modes by tuning the radius of the cavity holes. For the lower frequency even mode, a *Q*-factor as high as 13,360 was achieved when $R_{c}=0.455a$. The *Q*-factor of higher frequency even mode reached 8000 when $R_{c}=0.44a$. The *Q*-factor could be improved by adjusting $R_{c}$, and this allowed for the tuning of the resonance frequency of each mode. The resonant peak was pushed toward higher frequencies (shorter wavelengths) when $R_{c}$ was increased in the cavity, Figure 5b. 

To quantitatively analyze the sensitivity of the proposed sensor device, we observe the shift of the resonant peak when the refractive index of the sample (penetrating the holes of the photonic crystal) is altered. The sensitivity is in our case suitably defined as the ratio of the resonant wavelength change to the refractive index change.

(1) $$S=\frac{d\lambda }{dn}[\frac{nm}{RIU}]$$

**Figure 6:**
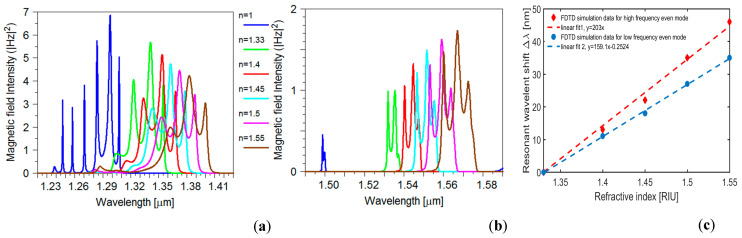
(**a**) Resonant wavelength of the high-frequency even mode for different refractive indices. (**b**) Transmission spectrum of the low-frequency even mode for different refractive indices. (**c**) The blue circles and red diamonds indicate the shift values of resonant wavelengths for different refractive index variations, and the dashed lines are linearly fit when $R_{c}=0.455a$. The expressions for the two linear fitting curves are y=203x and y=150.1x+0.2524 for the dashed red and blue lines, respectively.

The resonant wavelengths when there is no analyte in the holes and on the surface were obtained at 1239–1308 nm (Figure 6a) and 1498–1500 nm (Figure 6b) for higher and lower frequency modes respectively. After filling the holes with a sample with a refractive index of 1.33 resonant wavelengths shift to 1305–1356 nm and 1532–1537 nm respectively. The resonance wavelength shift for each mode is calculated based on these wavelengths. By further increasing the refractive index the resonant peaks shift to a higher wavelength range. The red diamonds and blue circles in Figure 6c indicate the shift values of resonant wavelengths for different refractive index alterations for higher and lower frequency even modes when $R_{c}=0.455a$, respectively. Based on these data, we can calculate sensitivity *S* of 203 nm/RIU (refractive index unit) for the higher energy even mode and 159 nm/RIU for the lower energy even mode. The designed optical biosensor can detect a variety of biological target liquids such as various cancerous cells in the human body [4,31]. 

Future work will address the fabrication of the device. Here, we have outlined a prospective fabrication process. The fabrication will utilize a silicon-on-insulator (SOI) wafer. The initial step will involve mask processing through photolithography to imprint the designed structure’s mask onto the resist. Subsequently, the top silicon (Si) layer will undergo vertical etching via a dry etching process to transfer the pattern onto the silicon. Reactive ion etching (RIE) is commonly used for this step. Lastly, the SiO_2_ layer will be eliminated in a wet etching process, in which a chemical solution will selectively remove the oxide.

## 4. Conclusions

In conclusion, this study presents a new sensor structure based on a two-dimensional photonic crystal composed of a hexagonal array of air holes in a silicon substrate, and its performance was validated by using the finite difference time domain (FDTD) method for detecting refractive index changes. The structure consisted of a PhC waveguide coupled to a coupled cavity waveguide (CCW) integrated within a slab waveguide. The dispersion diagram calculated with the plane wave expansion (PWE) method demonstrated the feasibility of the concept of coupling light from a photonic crystal waveguide to a coupled cavity waveguide with a proper symmetry profile. The potential of the structure as a sensor was demonstrated by studying the dispersion properties of the propagating modes. The radii of five holes in each coupled cavity were adjusted slightly to optimize the *Q*-factor of the structure. A simulated sensitivity of 203 nm/RIU and *Q*-factor of 13,360 was achieved for a wide range of refractive indices from 1.33 to 1.55. This design was particularly effective at detecting liquid biotargets. The design strategy involved avoiding sharp corners and small elements together with a short length. Such a system is fully compatible with advanced Si-based fabrication technologies, such as CMOS and micro-electro-mechanical systems (MEMS), so the device can be manufactured with standard fabrication processes.

## Data Availability

Data are contained within the article.

## References

[1] Kozma P., Kehl F., Ehrentreich-Förster E., Stamm C., Bier F.F. (2014). Integrated planar optical waveguide interferometer biosensors: A comparative review. Biosens. Bioelectron..

[2] Pergande D., Geppert T.M., Von Rhein A., Schweizer S.L., Wehrspohn R.B., Moretton S., Lambrecht A. (2011). Miniature infrared gas sensors using photonic crystals. J. Appl. Phys..

[3] Liu W., Liu X., Zhou J., Xu C., Dong B., Lee C. (2022). Larger-Than-Unity External Optical Field Con fi nement Enabled by Metamaterial-Assisted Comb Waveguide for Ultrasensitive Long-Wave Infrared Gas Spectroscopy. Nano Lett..

[4] Zadeh F.R., Kaatuzian H., Danaie M. Hybrid Photonic Crystal Cavity as a Sensitive Label-Free Biosensor. Proceedings of the 2019 27th Iranian Conference on Electrical Engineering (ICEE).

[5] Li J. (2020). A review: Development of novel fiber-optic platforms for bulk and surface refractive index sensing applications. Sens. Actuators Rep..

[6] Parandin F., Heidari F., Rahimi Z., Olyaee S. (2021). Two-Dimensional photonic crystal Biosensors: A review. Opt. Laser Technol..

[7] Pühringer G., Consani C., Jakoby B. (2020). Impact of Different Metals on the Performance of Slab Tamm Plasmon Resonators. Sensors.

[8] Saeidi P., Jakoby B., Pühringer G., Tortschanoff A., Stocker G., Dubois F., Spettel J., Grille T., Jannesari R. (2021). Designing Mid-Infrared Gold-Based Plasmonic Slot Waveguides for CO_2_-Sensing Applications. Sensors.

[9] Kita S., Hachuda S., Otsuka S., Endo T., Imai Y., Nishijima Y., Misawa H., Baba T. (2011). Super-sensitivity in label-free protein sensing using a nanoslot nanolaser. Opt. Express.

[10] Vasilantonakis N., Wurtz G.A., Podolskiy V.A., Zayats A.V. (2015). Refractive index sensing with hyperbolic metamaterials: Strategies for biosensing and nonlinearity enhancement. Opt. Express.

[11] He L., Yi Y., Zhang J., Xu X., Tang B., Li G., Zeng L., Chen J., Sun T., Yi Z. (2023). A four-narrowband terahertz tunable absorber with perfect absorption and high sensitivity. Mater. Res. Bull..

[12] Lai R., Chen H., Zhou Z., Yi Z., Tang B., Chen J., Yi Y., Tang C., Zhang J., Sun T. (2023). Design of a Penta-Band Graphene-Based Terahertz Metamaterial Absorber with Fine Sensing Performance. Micromachines.

[13] Wu F., Chen M., Xiao S. (2022). Wide-angle polarization selectivity based on anomalous defect mode in photonic crystal containing hyperbolic metamaterials. Opt. Lett..

[14] Kar C., Jena S., Udupa D.V., Rao K.D. (2023). Tamm plasmon polariton in planar structures: A brief overview and applications. Opt. Laser Technol..

[15] Wu F., Liu T., Xiao S. (2023). Polarization-sensitive photonic bandgaps in hybrid one-dimensional photonic crystals composed of all-dielectric elliptical metamaterials and isotropic dielectrics. Appl. Opt..

[16] Bikbaev R.G., Vetrov S.Y., Timofeev I.V. (2019). Epsilon-Near-Zero Absorber by Tamm Plasmon Polariton. Photonics.

[17] Baghdouche L.K., Cassan E. (2018). Mid-infrared refractive index sensing using optimized slotted photonic crystal waveguides. Photonics Nanostruct. Fundam. Appl..

[18] Painam B., Kaler R.S., Kumar M. (2016). Photonic Crystal Waveguide Biochemical Sensor for the Approximation of Chemical Components Concentrations. Plasmonics.

[19] Saker K., Bouchemat T., Lahoubi M., Bouchemat M., Pu S. (2019). Magnetic field sensor based on a magnetic-fluid-infiltrated photonic crystal L4 nanocavity and broadband W1 waveguide. J. Comput. Electron..

[20] Jannesari R., Grille T. Gas sensing with a high-quality-factor photonic crystal ring resonator. Proceedings of the SPIE—The International Society for Optical Engineering.

[21] Jannesari R., Pühringer G., Grille T., Jakoby B. (2020). Design and Analysis of a Slot Photonic Crystal Waveguide for Highly Sensitive Evanescent Field Absorption Sensing in Fluids. Micromachines.

[22] Karle T.J., Chai Y.J., Morgan C.N., White I.H., Krauss T.F. (2004). Observation of pulse compression in photonic crystal coupled cavity waveguides. J. Light. Technol..

[23] Sanchis P., García J., Martínez A., Martí J. (2005). Pulse propagation in adiabatically coupled photonic crystal coupled cavity waveguides. J. Appl. Phys..

[24] Danaie M., Geravand A., Mohammadi S. (2018). Photonic crystal double-coupled cavity waveguides and their application in design of slow-light delay lines. Photonics Nanostruct. Fundam. Appl..

[25] Huang L., Tian H., Zhou J., Liu Q., Zhang P., Ji Y. (2015). Label-free optical sensor by designing a high-Q photonic crystal ring-slot structure. Opt. Commun..

[26] Jannesari R., Grille T., Consani C., Stocker G., Tortschanoff A., Jakoby B. (2021). Design of a Curved Shape Photonic Crystal Taper for Highly Efficient Mode Coupling. Sensors.

[27] Johnson S.G., Bienstman P., Skorobogatiy M.A., Ibanescu M., Lidorikis E., Joannopoulos J.D. (2002). Adiabatic theorem and continuous coupled-mode theory for efficient taper transitions in photonic crystals. Phys. Rev. E Stat. Phys. Plasmas Fluids Relat. Interdiscip. Top..

[28] “RSoft’s Photonic Design Suite Version Synopsys RSoft 2019.09”, RSoft’s Photonic Design Suite. [Online]. https://www.synopsys.com/photonic-solutions.html.

[29] Sakoda K., Kawai N., Ito T., Chutinan A., Noda S., Mitsuyu T., Hirao K. (2001). Photonic bands of metallic systems. I. Principle of calculation and accuracy. Phys. Rev. B Condens. Matter Mater. Phys..

[30] Pergande D., Von Rhein A., Geppert T.M., Wehrspohn R.B. (2009). Coupling schemes for low-group velocity photonic crystal devices. J. Comput. Theor. Nanosci..

[31] Vikas, Saccomandi P. (2023). Antimonene-Coated Uniform-Waist Tapered Fiber Optic Surface Plasmon Resonance Biosensor for the Detection of Cancerous Cells: Design and Optimization. ACS Omega.

